# Upregulation of ATG9b by propranolol promotes autophagic cell death of hepatic stellate cells to improve liver fibrosis

**DOI:** 10.1111/jcmm.18047

**Published:** 2023-11-16

**Authors:** Sining Wang, Qian Ding, Aiyuan Xiu, Yifu Xia, Guangchuan Wang, Chunqing Zhang

**Affiliations:** ^1^ Department of Gastroenterology Shandong Provincial Hospital, Cheeloo College of Medicine, Shandong University Jinan China; ^2^ Department of Gastroenterology Shandong Provincial Hospital Affiliated to Shandong First Medical University Jinan China

**Keywords:** ATG9b, autophagy, hepatic stellate cells, liver cirrhosis, portal hypertension

## Abstract

Proranolol has long been recommended to prevent variceal bleeding in patients with cirrhosis. However, the mechanisms of propranolol in liver fibrosis have not yet been thoroughly elucidated. Autophagic cell death (ACD) of activated hepatic stellate cells (HSCs) is important in the alleviation of liver fibrosis. Our study aims to assess the mechanisms of propranolol regulating HSC ACD and liver fibrosis. ACD of HSCs was investigated using lentivirus transfection. The molecular mechanism was determined using a PCR profiler array. The role of autophagy‐related protein 9b (ATG9b) in HSC ACD was detected using co‐immunoprecipitation and co‐localization of immunofluorescence. Changes in the signalling pathway were detected by the Phospho Explorer antibody microarray. Propranolol induces ACD and apoptosis in HSCs. ATG9b upregulation was detected in propranolol‐treated HSCs. ATG9b upregulation promoted ACD of HSCs and alleviated liver fibrosis in vivo. ATG9b enhanced the P62 recruitment to ATG5‐ATG12‐LC3 compartments and increased the co‐localization of P62 with ubiquitinated proteins. The PI3K/AKT/mTOR pathway is responsible for ATG9b‐induced ACD in activated HSCs, whereas the p38/JNK pathway is involved in apoptosis. This study provides evidence for ATG9b as a new target gene and propranolol as an agent to alleviate liver fibrosis by regulating ACD of activated HSCs.

## 1 INTRODUCTION

Liver fibrosis is characterised by excessive extracellular matrix (ECM) formation, which can lead to liver cirrhosis and even liver failure. Liver cirrhosis is the 11th most common cause of death worldwide and accounts for approximately one million deaths worldwide annually.^1^, ^2^, ^3^ Previous studies emphasised the crucial role of cell death during liver cirrhosis.^4^, ^5^ Upon liver injury, activated hepatic stellate cells (HSCs) can accumulate ECM and secrete cytokines and chemokines, which are critical effectors of the fibrotic response. Consequently, deactivation and clearance of activated HSCs are very vital in treating liver fibrosis.^6^ However, the mechanisms underlying the clearance of activated HSCs need to be better characterised.

Although autophagy was characterised initially as a cytoprotective mechanism that balances the generation of cellular constituents and breakdown of damaged organelles, there is more and more evidence for a novel form of cell death in mammalian cells, namely autophagic cell death (ACD).^7^, ^8^, ^9^, ^10^, ^11^, ^12^ Studies in mammals and other models have verified that ACD occurs under specific developmental or pathophysiological conditions.^13^ However, the molecular mechanism of ACD in HSCs is still unclear.

Autophagic cell death mechanistically depends on the autophagic machinery or components thereof,^14^ so ACD can be modulated by autophagy‐related (*ATG*) genes.^13^, ^15^, ^16^ Liu et al.^12^ showed that cell death with morphological characteristics of autophagy could be waned by ATG13 or ATG14 knockdown. Moreover, the accumulation of autophagosomes and upregulation of ATG5 were accompanied by subsequent ACD in fibroblast cell lines.^11^ Thus, regulating the ACD of activated HSCs via *ATG genes* may cause HSC death and serve as a new approach for the alleviation of liver fibrosis. The role of autophagy‐related protein 9b (ATG9b) in the ACD of HSC and liver fibrosis has not been revealed before. This study aimed to identify the effect of ATG9b on HSC death and liver fibrosis.

Interestingly, we also discovered propranolol could induce cell death in HSCs via ACD in this study. Propranolol has long been recommended in primary and secondary prevention of variceal bleeding in patients with cirrhosis because it can decrease splanchnic blood flow.^17^, ^18^ In addition to directly reducing portal blood flow, propranolol may exert the potential beneficial effect of decreasing vascular resistance in the liver by alleviating liver fibrosis.^18^, ^19^ Therefore, it is of great necessity to explore the role and mechanisms of propranolol in liver fibrosis.

In the present study, we emphasised that ACD was involved in the propranolol‐induced death of activated HSCs. Our observations also revealed the role and underlying mechanisms of ATG9b in regulating ACD of activated HSCs to alleviate liver fibrosis.

## 2 MATERIALS AND METHODS

### 2.1 Reagents and antibodies

Dulbecco's modified Eagle's medium (DMEM) and foetal bovine serum (FBS) were obtained from Gibco. Earle's balanced salt solution (EBSS) and propranolol were purchased from Sigma‐Aldrich. Rapamycin (RAPA), 3‐methyladenine (3‐MA) and chloroquine (CQ) were obtained from MedChemExpress, and ZVAD‐FMK, necrostatin‐1, liproxstatin‐1, SB203580, SP600125, SC79 and 740Y‐P were purchased from SelleckChem. Then, these reagents were dissolved in dimethyl sulfoxide (DMSO) (MP Biomedicals or water and stored at −80°C. Primary antibodies against Akt, p‐Akt, p38 MAPK, p‐p38 MAPK, JNK, p‐JNK, Erk1/2 and p‐Erk1/2 were purchased from Cell Signalling Technology. Primary antibodies against alpha‐smooth muscle actin (α‐SMA), fibronectin (FN), LC3B, P62, p‐PI3K p85, PI3K p85, ATG9b, ATG9a, mTOR, p‐mTOR, ATG12, ATG5 and anti‐ubiquitin were procured from Abcam. Primary antibodies against beta‐actin, alpha‐tubulin, GAPDH and HRP‐conjugated secondary antibodies were obtained from Proteintech. DyLight 549‐conjugated and DyLight 488‐conjugated secondary antibodies were provided by Abbkine.

### 2.2 Mouse experiments

Six‐week‐old male C57BL/6 mice (Vital River Company) were fed in a suitable environment at the Animal Center of Shandong Provincial Hospital, Shandong University. Mice were administered AAV8‐shATG9b, AAV8‐OE‐ATG9b, or AAV8‐Ctrl (Genechem) twice a week through the tail vein.

To establish the liver fibrosis model, we mixed CCl_4_ with olive oil (1:4) and intraperitoneally injected the mixture (0.5 mL/100 g) into mice twice weekly. After 2 weeks, propranolol (10 mg/kg) or water was administered daily by gavage for 6 weeks.

### 2.3 Cell culture and transfection

The rat HSC cell line T6 was cultured in a complete DMEM medium. The human HSC cell line LX2 and normal human liver cell line HL‐7702 were cultured in a complete 1640 medium. According to the manufacturer's instructions, control siRNA and ATG9b‐siRNAs (Genomeditech) of corresponding species were transfected into LX2 or T6 cells at 50 pmol (12‐well plate) using Lipofectamine 3000 (Life Technologies) for in vitro knockdown. The target interfering sequences are represented in Table 1. For overexpression of ATG9b in vitro, LX2 or T6 cells were transfected with the ATG9b plasmid and control vector plasmid (GeneChem) of the corresponding species. To detect autophagic flux, we used GFP‐LC3B adenovirus (Hanbio) and mCherry‐EGFP‐LC3B lentivirus (Genomeditech) to transfect into LX2 or T6 cells, followed by observation under a laser confocal microscopy (Leica TCS SP8).

**TABLE 1:** Target sequences for RNAi.

Species	Gene	Target sequence (5′‐3′)
Human	ATG9b‐siRNA1	GGCUCAACCUGCAAUGACATT
		UGUCAUUGCAGGUUGAGCCTT
Human	ATG9b‐siRNA2	GAUCCCUGAACAGGAUUAUTT
		AUAAUCCUGUUCAGGGAUCTT
Human	ATG9b‐siRNA3	UCACCAAGAUCUACAGCUATT
		UAGCUGUAGAUCUUGGUGATT
Rat	ATG9b‐siRNA1	UCACCAAGAUCUACAGCUATT
		UAGCUGUAGAUCUUGGUGATT
Rat	ATG9b‐siRNA2	GUCCUAAGUUUCUUGGACATT
		UGUCCAAGGAACUUAGAGCTT
Rat	ATG9b‐siRNA3	GAGAAGCCGUCUUGGUCAATT
		UUGACCAAGACGGCUUCUCTT

### 2.4 RNA isolation, quantitative real‐time PCR (qRT‐PCR) and PCR array sequencing of RNA (RNA‐seq)

Total RNA was isolated from LX2 and T6 cells utilizing TRIzol reagent; then, a reverse transcription kit was used to synthesize cDNA (Takara). A SYBR Green PCR kit (Takara) was used for qRT‐PCR. The mRNA levels were normalised to those of GAPDH. The primers used are represented in Table 2. RNA‐seq of T6 cells was performed by Lianchuan Biotechnology Co., Ltd.

**TABLE 2:** Primers for qRT‐PCR.

Species	Primer	Forward	Reverse
Human	α‐SMA	ATTGCCGACCGAATGCAGA	ATGGAGCCACCGATCCAGAC
Human	FN	GATAAATCAACAGTGGGAGC	CCCAGATCATGGAGTCTTTA
Human	Collagen	TAGGGTCTAGACATGTTCAGCTTT	CGTTCTGTACGCAGGTGATTG
Human	ATG9b	CAAGACTCACCCATCCACGG	GTGGTAGCTGTAGATCTTGGTG
Human	ATG9a	TCTGCGCATCCCTATGTCTG	GGAGGATGCGGTGGTAGATG
Human	GAPDH	CCAGGGCTGCTTTTAACTCT	GGACTCCACGACGTACTCA
Rat	α‐SMA	TGGAAAAGATCTGGCACCAC	TCCGTTAGCAAGGTCGGATG
Rat	FN	AAGCTACCATTCCAGGCCAC	GTCACTTCTTGGTGCCCGTA
Rat	Collagen	TGACTGGAAGAGCGGAGAGTA	TGCAGTAGACCTTGATGGCA
Rat	ATG9b	GCTGCTAGTCCTCACCATCT	CCTCTGGAATGAAGGACCTGG
Rat	ATG9a	GACACTGAATACCAGCGCCTA	GGTGCCAAGGTGATTTGCTC
Rat	GAPDH	GGCACAGTCAAGGCTGAGAATG	ATGGTGGTGAAGACGCCAGTA

### 2.5 Cell Counting Kit 8 (CCK8) assay

LX2, T6 and HL‐7702 cells, with or without transfection, were cultured in 96‐well plates overnight. After cells were treated with different reagents for 24 h, phosphate‐buffered saline (PBS) was used to wash the cells, and 10 μL CCK8 solution (Vazyme) was added to each well. In an hour, the optical density (OD) values were measured at 450 nm by using a microplate reader (Bio‐Rad Model 550).

### 2.6 Phospho‐protein profiling by phospho‐antibody array

T6 cells treated with the ATG9b plasmid and control vector were harvested and detected using the Phospho Explorer Antibody Array (Lianchuan Biotechnology Co., Ltd.). The analysis of the phosphorylation ratio in different groups was performed as previously described.^20^

### 2.7 Co‐immunoprecipitation (Co‐IP) and immunoblotting assay

Briefly, IP lysis buffer (Beyotime) was used to lyse LX2 cells from different groups for 30 min. Then collected buffer was centrifuged at 14,000 × *g* for 30 min at 4°C. A 10% supernatant was collected for immunoblotting analysis, and the remaining supernatant was added to 1 μg corresponding antibody and protein A/G beads with agarose (sc‐2003; Santa Cruz) overnight at 4°C with slow stagger. On the second day, beads were acquired after centrifugation at 2500 × *g* for 3 min at 4°C. The beads were washed with 1 mL lysis buffer three times. In the end, we put 15 μL loading buffer into the beads, and the beads were boiled for 5 min for subsequent immunoblotting. The process of immunoblotting assay was as previously described.^21^

### 2.8 Immunofluorescent staining

For immunofluorescence staining, 4% paraformaldehyde was used to fix HSCs or slides of mouse liver tissues for 15 min, and 5% BSA was used to block HSCs or slides for 1 h at room temperature. Subsequently, corresponding primary antibodies were added to HSCs or slides at 4°C. On the second day, fluorescently labelled secondary antibodies were added and incubated for 90 min at 37°C, and DAPI was used for 10 min. Finally, a laser confocal microscope was used to acquire images.

### 2.9 Transmission electron microscopy (TEM)

LX2 cells were treated with 80 μM propranolol or DMSO, whereas T6 cells were treated with 50 μM propranolol or DMSO for 24 h. We fixed cells with 2% glutaraldehyde and 1% osmium tetroxide and performed graded dehydration. Subsequently, we made ultrathin and stained slices with 1% lead citrate and 10% uranium lactate. Images were obtained through a transmission electron microscope (HT7800; Hitachi).

### 2.10 Transwell migration assay

LX2 or T6 cells (1 × 10^4^ cells/well) were seeded in the upper chambers of transwell plates, following medium containing 10% FBS in the lower rooms. Haematoxylin–eosin (HE) staining was performed to label migrated cells on the lower surface of the chamber. We observed stained cells under a microscope (Olympus).

### 2.11 Gel contraction assay

Collagen gels (1 mg/mL) made by type I rat‐tail collagen (BD Bioscience), 10 × PBS, and 0.1 mol/L NaOH were added into a 24‐well culture plate (0.5 mL/well). After 1 h, collagen gel was gelated and then exposed to a complete culture medium (0.5 mL/well) overnight at 37°C. On the second day, cells were seeded into the plates. After treatment with different reagents, collagen gels were separated from the side wall and incubated for another 24 h at 37°C. Each gel was photographed.

### 2.12 Detection of apoptosis using TUNEL staining and flow cytometry

We detected apoptosis using one‐step TUNEL Apoptosis Assay Kit (Beyotime) according to the manufacturer's instructions, and fluorescence was examined under a laser confocal microscope (TCS SP8; Leica).

We also used Apoptosis Detection Kit I (BD Biosciences) to examine apoptosis according to the manufacturer's protocols and analyse results by flow cytometry (FACS Calibur Flow Cytometer, BD Biosciences) within 15 min.

### 2.13 Primary HSC isolation

Primary stellate cells from mouse livers were isolated according to a previously published protocol.^22^

### 2.14 Histological analyses, immunohistochemical staining and blood analysis

Briefly, the liver tissues were fixed in tissue fixation fluid for 24 h and embedded in paraffin blocks. Then, tissue slides of serial 5‐μm‐thick sections were stained with HE, Sirius red and Masson for histological analysis. According to the manufacturer's instructions for DAB chromogenic reagent, some slides were incubated with the appropriate primary antibodies. Alanine aminotransferase (ALT) and aspartate aminotransferase (AST) levels in the mouse serum were measured using an automatic biochemical analyzer (Chemray 800; Rayto) according to the manufacturer's instructions.

### 2.15 Statistical analysis

Data that occurred in this study was expressed as the mean ± standard deviations (SDs). Differences were analysed using Student's *t*‐test or one‐way analysis of variance (*p < 0.05, **p < 0.01, ***p < 0.001, ****p < 0.0001) using GraphPad Prism 9 (GraphPad Software, Inc.).

## 3 RESULTS

### 3.1 Propranolol inhibits the viability and activation of HSCs

**FIGURE 1:**
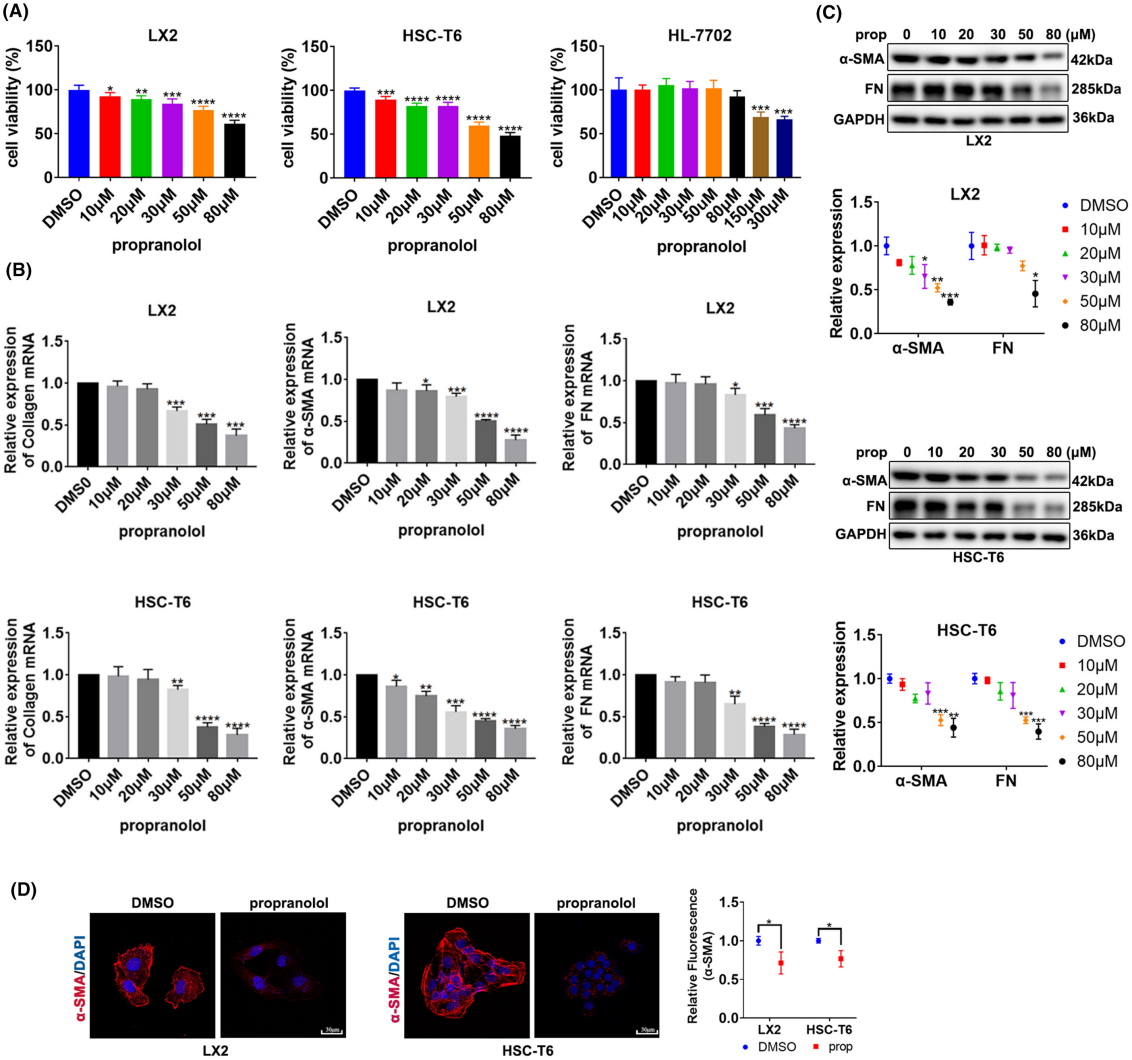
Propranolol inhibits the viability and activation of HSCs. (A) Viability of HSC cell lines LX2 and T6 and normal liver cell line HL‐7702 treated with different concentrations of propranolol. (B, C). LX2 and T6 cells were treated with different concentrations of propranolol or DMSO for 24 h, and the levels of α‐SMA, collagen and fibronectin (FN) were determined. (D) LX2 cells was treated with 80 μM propranolol or DMSO, and T6 cells were treated with 50 μM propranolol or DMSO; α‐SMA were revealed by immunofluorescence. Data are presented as mean ± standard error from three independent experiments (*p < 0.05, **p < 0.01, ***p < 0.001, ****p < 0.001).

To detect the cytotoxic effect of propranolol, we used CCK8 assays to detect cell viability. The viability of propranolol‐treated LX2 and T6 cells was significantly decreased in a dose‐dependent manner. In contrast, that of HL‐7702 cells (the normal liver cell line) showed only a slight decrease when propranolol concentration was 300 μM (Figure 1A). Above results reveal that propranolol inhibits the proliferation and viability of HSCs but not of normal liver cells.

We examined the degree of HSC activation. After treatment with propranolol, the levels of α‐SMA, Collagen and FN decreased in LX2 and T6 cells (Figure 1B,C). Moreover, we chose 50 μM propranolol for further investigation in T6 cells and 80 μM propranolol in LX2 cells. Immunofluorescence analysis of α‐SMA further confirmed that propranolol inhibited HSC activation (Figure 1D).

### 3.2 Propranolol induces ACD and apoptosis in HSCs

**FIGURE 2:**
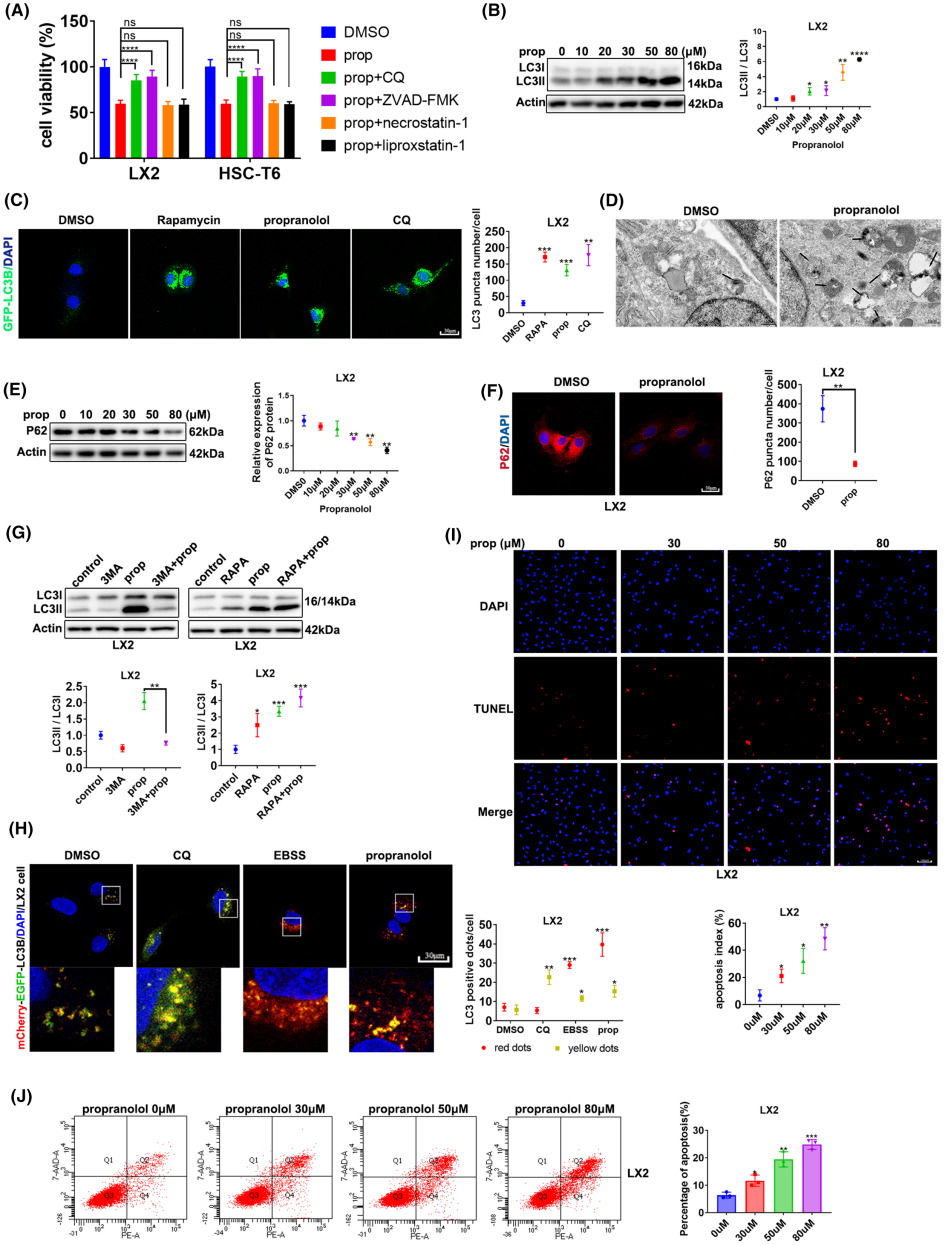
Propranolol induces ACD and apoptosis in HSCs. (A) LX2 and T6 cells were treated with propranolol, with or without the indicated inhibitors (CQ, 30 μM; ZVAD‐FMK, 10 μM; necrostatin‐1, 10 μM; liproxstatin‐1, 100 nM) for 24 h. Cell viability was assessed. (B) LX2 cells were treated with increasing concentrations of propranolol for 24 h, and LC3II accumulation was determined using western blotting. (C) LX2 cells expressing GFP‐LC3B (green) were treated with DMSO, chloroquine (CQ) (30 μM), rapamycin (100 nM), or propranolol for 24 h. Images of GFP‐LC3B puncta were acquired using confocal microscopy. (D) Representative transmission electron microscopy pictures of autophagosomes/autolysosomes (arrows) in LX2 cells incubated with propranolol or DMSO for 24 h. (E, F) P62 accumulation in LX2 cells was assessed using western blotting and immunofluorescence. (G) LX2 cells were treated with DMSO, 3‐MA (10 mM), or rapamycin (RAPA), alone or in the presence of propranolol. The conversion of LC3I to LC3II was determined using western blotting. (H) Representative images of autophagosomes (yellow dots generated from the overlap of mCherry and EGFP puncta) and autolysosomes (red dots generated from mCherry puncta) after 24 h of treatment with EBSS, 30 μM CQ, propranolol or DMSO in LX2 cells transfected with the mCherry‐EGFP‐LC3B‐Lentivirus. (I, J) LX2 cells were treated with different concentrations of propranolol for 24 h, and apoptosis was assessed using TUNEL staining and flow cytometry. Data are presented as mean ± standard error from three independent experiments (*p < 0.05, **p < 0.01, ***p < 0.001, ****p < 0.001; ns, not significant).

To determine the cell death mode affecting HSC activity, we used various cell death inhibitors. Propranolol‐mediated cell death in LX2 and T6 cells was prevented by CQ (a potent autophagy inhibitor) and ZVAD‐FMK (a potent apoptosis inhibitor), but not by necrostatin‐1 (a potent necroptosis inhibitor) or liproxstatin‐1 (a potent ferroptosis inhibitor) (Figure 2A). These results indicate that ACD and apoptosis participate in propranolol‐induced cell death in HSCs.

We examined autophagosome formation and autophagic flux to verify whether ACD participates in propranolol‐induced cell death. Immunoblotting results showed that propranolol enhanced LC3II/I levels in HSCs (Figure 2B and Figure S1A). The number of GFP‐LC3B puncta increased after propranolol treatment compared with treatment with DMSO as the negative control and RAPA and CQ as positive controls (Figure 2C and Figure S1B). Furthermore, the result of TEM showed that propranolol‐treated cells formed more autophagic vacuoles than the DMSO‐treated cells (Figure 2D and Figure S1C).

We further explored the process of autophagy flux. We observed that the amount of P62 decreased (Figure 2E,F and Figure S1D,E), demonstrating increased degradation of autophagic lysosomes and indicating that increased autophagosomes are not caused by inhibition of late‐stage autophagy. Immunoblotting assays showed that co‐incubation of propranolol with autophagy inhibitors (3‐MA) significantly decreased the effect of propranolol on the expression of LC3II, while co‐incubation of propranolol with RAPA did not (Figure 2G and Figure S1F). We then used a type of mCherry‐EGFP‐LC3B‐Lentivirus to transfect into HSCs. The number of yellow dots increased when autophagic flux was obstructed, and the quantity of yellow and red dots increased when autophagic flux was upregulated. The images of Figure 2H and Figure S1G showed propranolol treatment increased yellow and red dots than the DMSO treatment, consistent with the results of EBSS treatment. Conversely, the CQ treatment only increased the number of yellow dots than the DMSO treatment. The above results suggest that propranolol promotes autophagic flux.

To determine whether propranolol leads to apoptosis in HSCs, we performed a TUNEL assay and flow cytometry. Compared with those treated with DMSO, cells treated with propranolol had higher apoptosis ratios (Figure 2I,J and Figure S1H,I). Thus, propranolol induces apoptosis in HSCs.

### 3.3 ATG9b was upregulated in propranolol‐treated HSCs

**FIGURE 3:**
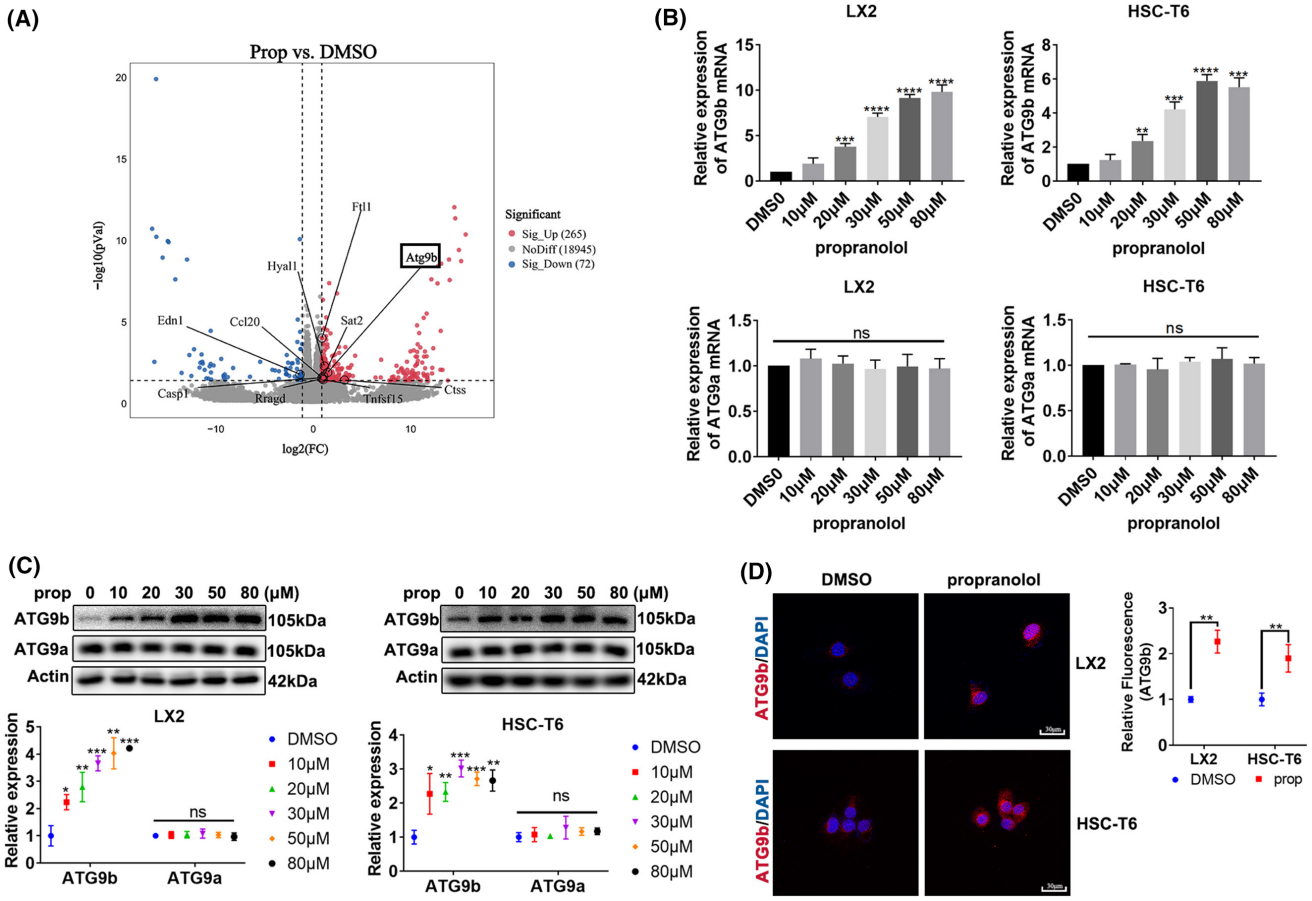
ATG9b was upregulated in propranolol‐treated HSCs. (A) T6 cells were treated with propranolol or DMSO for 24 h, and total RNA was extracted for RNA‐Seq. Hierarchical cluster analyses of significantly differentially expressed mRNAs: bright blue, underexpression; grey, no change; bright red, overexpression. (B, C) LX2 and T6 cells were treated with different concentrations of propranolol for 24 h; the mRNA and protein levels of ATG9b and ATG9a were then determined. (D) LX2 cells were treated with 80 μM propranolol or DMSO, and T6 cells were treated with 50 μM propranolol or DMSO; ATG9b levels were revealed using immunofluorescence. Data are presented as mean ± standard error from three independent experiments (*p < 0.05, **p < 0.01, ***p < 0.001, ****p < 0.001. ns; not significant).

To further explore the molecular mechanism of propranolol‐induced cell death, we performed RNA‐seq and analysed the mRNA expression of the cell death pathways (Figure 3A). We identified a crucial target gene, *ATG9b*, which was upregulated in propranolol‐treated T6 cells (Figure 3A). We verified the results that propranolol remarkably increased the expression of ATG9b without significant differences in ATG9a expression in LX2 and T6 cells (Figure 3B,C,D).

### 3.4 Upregulation of ATG9b mitigates activation, migration and contraction of HSCs

**FIGURE 4:**
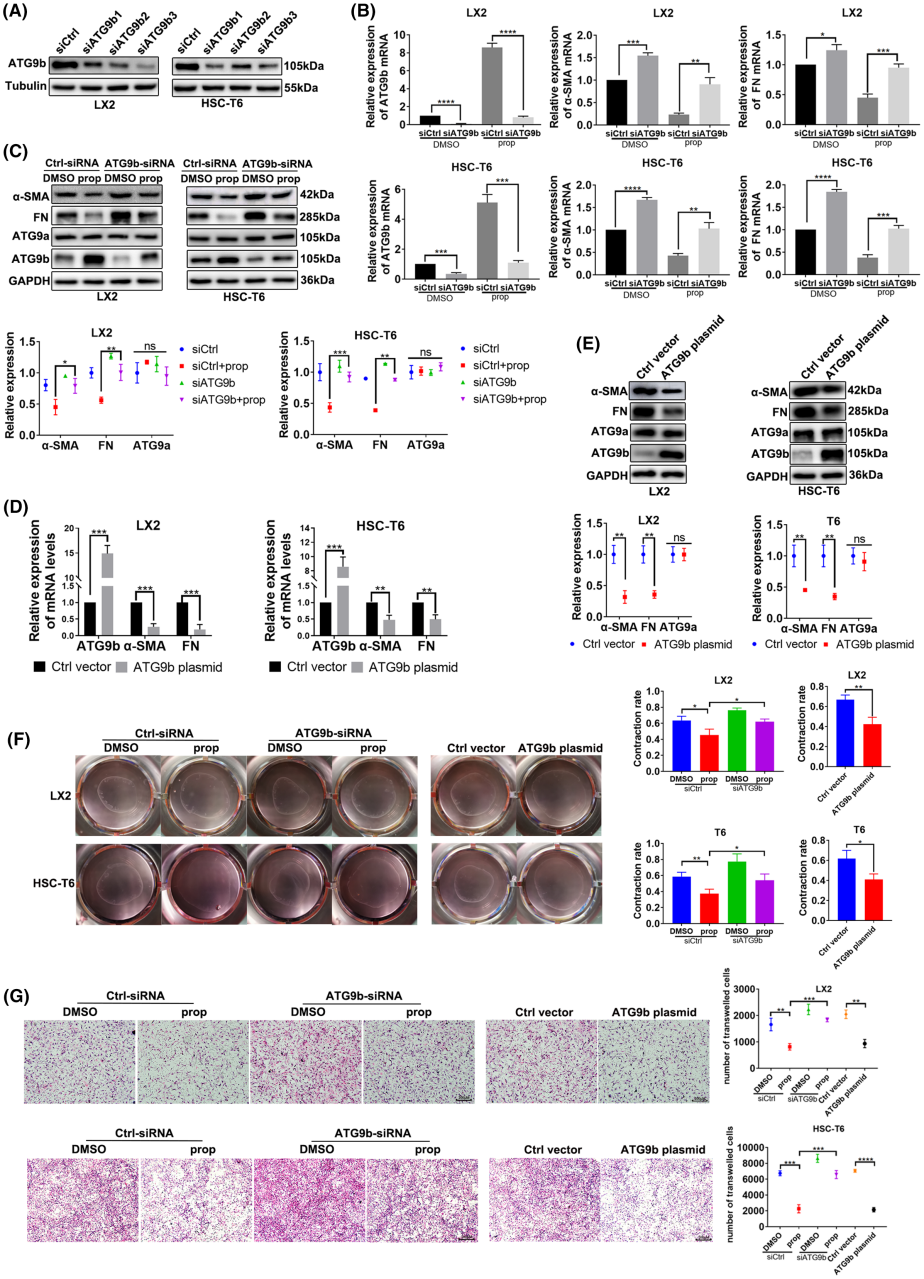
Upregulation of ATG9b mitigates activation, migration and contraction of HSCs. (A) LX2 and T6 cells were transfected with three ATG9b‐siRNAs or Ctrl‐siRNA of the corresponding species. Immunoblotting results of ATG9b in LX2 and T6 cells. (B–E) LX2 and T6 cells transfected with ATG9b‐siRNA or Ctrl‐siRNA were treated with propranolol or DMSO for 24 h; another group of LX2 and T6 cells were transfected with ATG9b plasmid or control vector. ATG9b, ATG9a, α‐SMA and fibronectin (FN) mRNA and protein levels were determined. (F) Gel contraction assays examined HSC contraction. (G) Transwell assays detected the migration ability of HSCs. Data are presented as mean ± standard error from three independent experiments (*p < 0.05, **p < 0.01, ***p < 0.001, ****p < 0.001; ns, not significant).

We investigated the role of ATG9b in HSCs. As shown in Figure 4A, ATG9b decreased after knockdown by ATG9b‐siRNAs in LX2 and T6 cells. The most efficient siRNA in LX2 and T6 cells were selected. RT‐PCR and immunoblotting assays showed that propranolol‐induced inhibition of α‐SMA and FN expression was counteracted by ATG9b knockdown in both LX2 and T6 cells, suggesting that propranolol inhibits HSCs activation by ATG9b (Figure 4B,C). Gel contraction and transwell assays showed that the propranolol‐induced inhibitory influence on contraction and migration was eliminated by the knockdown of ATG9b in LX2 and T6 cells (Figure 4F,G). To further determine whether ATG9b could alleviate HSCs activation, contraction and migration in a direct manner, LX2 and T6 cells were transfected with lentivirus to overexpress ATG9b. LX2 and T6 cells overexpressing ATG9b showed lower expression of α‐SMA and FN (Figure 4D,E) and lower contraction and migration ability (Figure 4F,G). In summary, our results showed that ATG9b participates in propranolol‐induced activation, migration and contraction of HSCs and that ATG9b independently modulates activation, migration and contraction of HSCs.

### 3.5 ATG9b regulates ACD and apoptosis of HSCs

**FIGURE 5:**
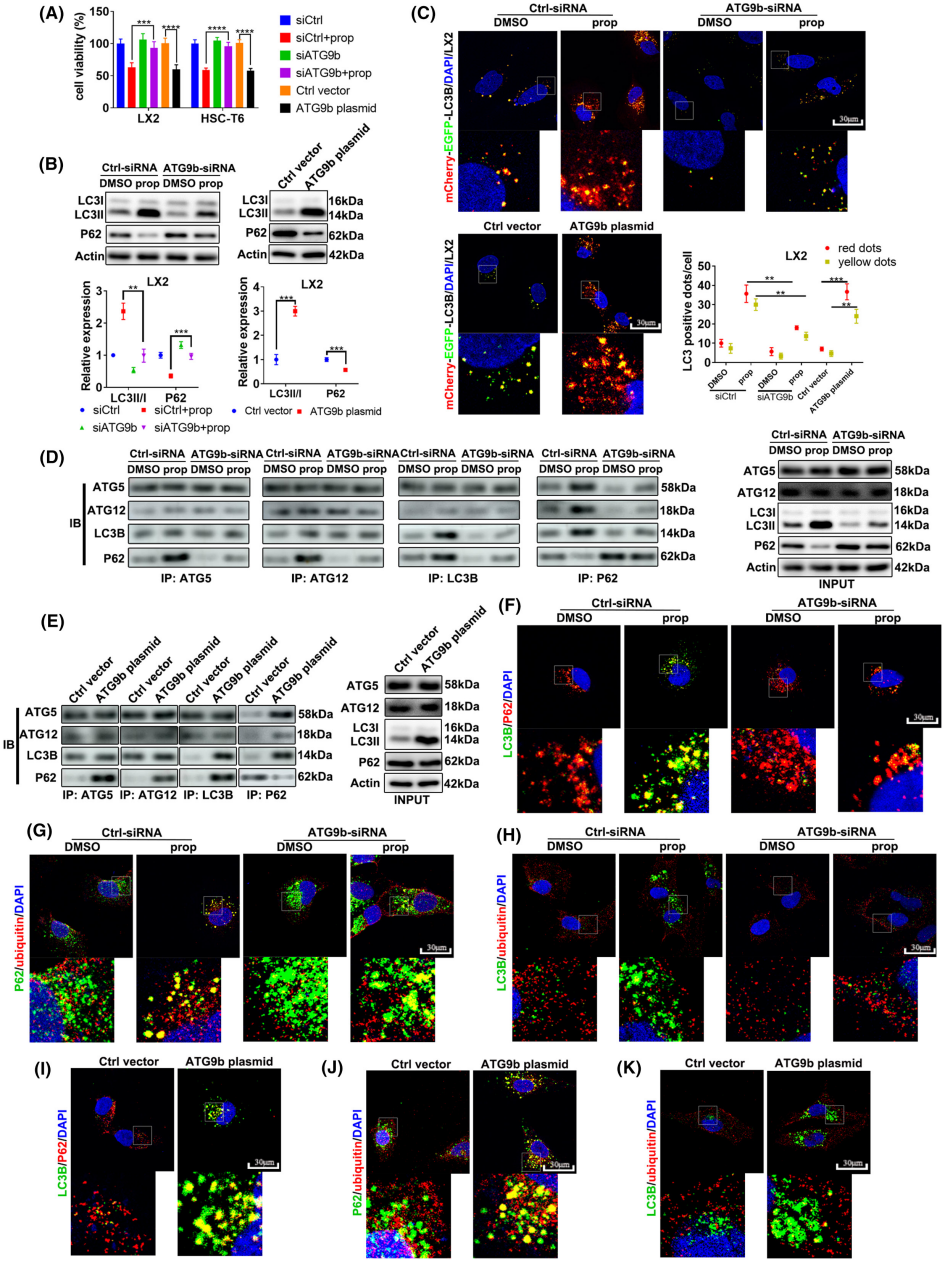
ATG9b regulates ACD of HSCs. LX2 and T6 cells were transfected with ATG9b‐siRNAs or Ctrl‐siRNA and co‐incubated with propranolol or DMSO for 24 h. Another group of LX2 and T6 cells were transfected with the ATG9b plasmid or control vector plasmid. (A) Cell viability was measured using the CCK8 assay. (B) The protein levels of LC3B and P62 in LX2 cells were detected using western blotting. (C) Autophagy flux in LX2 cells was detected using mCherry‐EGFP‐LC3B‐Lentivirus transfection. (D, E) Association of P62 with proteins forming autophagic vacuoles was measured using Co‐IP in LX2 cells. (F–K) Co‐localization of P62 with LC3B or ubiquitinated proteins was observed using immunofluorescence in LX2 cells. (**p < 0.01, ***p < 0.001, ****p < 0.001).

We assessed whether ATG9b contributes to the regulation of HSCs viability by CCK8. The findings indicate that ATG9b knockdown rescued propranolol‐caused cell death in HSCs, and ATG9b overexpression promoted cell death in HSCs (Figure 5A). This implies that ATG9b upregulation induces cell death of HSCs.

To distinguish the function of ATG9b in the ACD of HSCs, we detected LC3II expression by immunoblotting and observed autophagy flux by mCherry‐EGFP‐LC3B‐Lentivirus transfection. Knockdown of ATG9b blocked the propranolol‐induced enhanced expression of LC3II/I and reduced P62 expression (Figure 5B and Figure S2A); siATG9b‐HSCs treated with propranolol resulted in a decrease in both the number of yellow and red dots, compared with treatment with propranolol alone (Figure 5C and Figure S2C). The level of LC3II/I improved, and P62 accumulation was reduced in response to ATG9b overexpression in HSCs (Figure 5B and Figure S2B). HSCs overexpressing ATG9b showed more yellow and red dots than those in the control group (Figure 5C and Figure S2C). Taken together, ATG9b plays a crucial role in propranolol‐induced ACD of HSCs, and upregulating ATG9b without propranolol treatment also results in the ACD of HSCs.

To further explore which process of autophagy was influenced by propranolol and ATG9b in HSCs, we performed endogenous Co‐IP assays in LX2 cells. The recruitment of ATG5‐ATG12‐LC3 showed no changes in the propranolol treatment, siATG9b and siATG9b + propranolol groups compared with the DMSO group. Propranolol caused more interactions between P62 and the ATG5‐ATG12‐LC3 particle in LX2 cells; however, ATG9b knockdown abolished this effect (Figure 5D). ATG9b‐overexpressed LX2 cells showed more interactions between P62 and the ATG5‐ATG12‐LC3 particle than the control group (Figure 5E), which was further verified by that LC3B and P62 proteins were more co‐localised in propranolol‐treated LX2 cells or ATG9b‐overexpressed LX2 cells (Figure 5F,I).

Since P62 may act as an intermediate medium between LC3B and ubiquitinated protein in the late stage of autophagy flux,^23^ we examined whether ATG9b could affect the association of the protein complex. Inhibition of ATG9b reduced propranolol‐induced co‐localization of ubiquitinated substrates with P62 in LX2 cells, whereas its overexpression enhanced the co‐localization of ubiquitinated substrates with P62 in LX2 cells (Figure 5G,J). However, ATG9b did not affect the co‐localization of LC3B and ubiquitinated substrates (Figure 5H,K).

We examined whether ATG9b participates in apoptosis in HSCs. Propranolol‐induced apoptosis ratio of HSCs was eliminated following ATG9b knockdown, and HSCs overexpressing ATG9b showed an increased apoptosis ratio (Figure S3A–D). Consistent with the in vitro experimental results, ATG9b knockdown reversed propranolol‐induced apoptosis of HSCs, and ATG9b upregulation resulted in apoptosis of HSCs in cirrhotic mouse (Figure S3E). These results indicate that ATG9b modulates apoptosis of HSCs.

### 3.6 ATG9b mediates ACD by inhibiting PI3K/AKT/mTOR pathway and regulates apoptosis by activating p38/JNK pathway in HSCs

**FIGURE 6:**
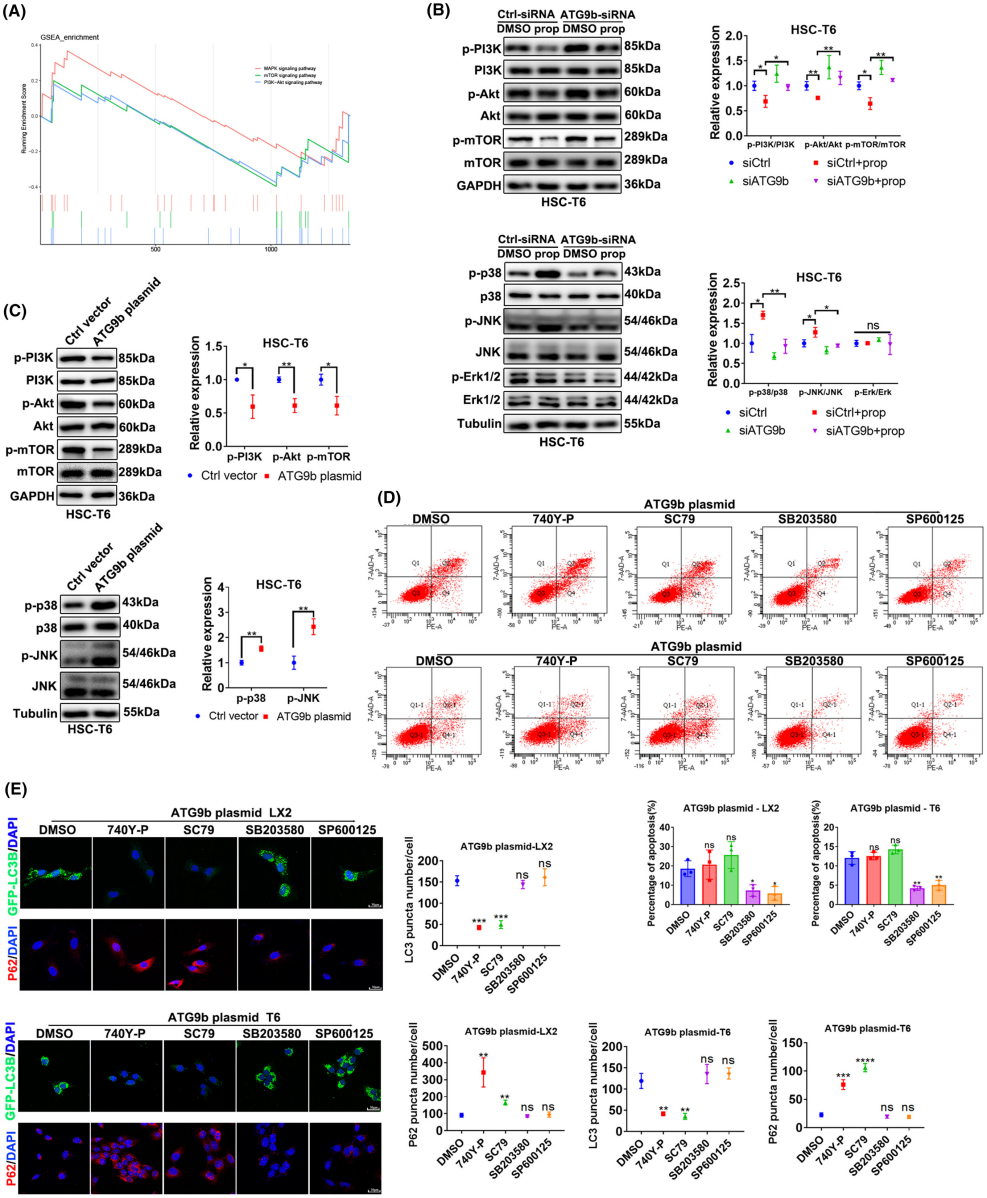
ATG9b mediates ACD by inhibiting the PI3K/AKT/mTOR pathway and regulates apoptosis by activating the p38/JNK pathway in HSCs. (A) GSEA analysis of pathways derived from T6 cells following treatment with ATG9b plasmid vs. control vector plasmid. (B, C) T6 cells were transfected with ATG9b‐siRNAs or Ctrl‐siRNA and co‐incubated with propranolol or DMSO for 24 h. Another group of T6 cells were transfected with ATG9b plasmid and control vector plasmid. The protein levels of p‐PI3K, PI3K, p‐AKt, Akt, p‐mTOR, mTOR, p‐p38, p38, p‐ERK, ERK, p‐JNK and JNK were detected. (D, E) LX2 and T6 cells overexpressing ATG9b were treated with SP600125 or SB203580 or SC79 or 740Y‐P. Apoptosis was measured using flow cytometry; GFP‐LC3B puncta and P62 expression were observed using confocal microscopy. Data are presented as mean ± standard error from three independent experiments (*p < 0.05, **p < 0.01, ***p < 0.001, ****p < 0.001; ns, not significant).

To investigate the mechanisms by which ATG9b regulates cell death in HSCs, we performed a Phospho Explorer antibody microarray to explore phosphorylation events in T6 cells overexpressing ATG9b. We analysed these potential pathways using KEGG and pathway mapping analyses. As shown in Figure 6A and Figure S4A, the PI3K‐AKT‐mTOR signalling pathways were significantly inhibited in the ATG9b‐overexpressed group, and MAPK signalling pathways were significantly overactivated in the ATG9b‐overexpressed group. Therefore, we examined the intermediates of the vital pathway by immunoblotting assays in T6 cells (Figure 6B,C).

To further verify the role of these signalling pathways in the ACD and apoptosis of HSCs induced by ATG9b, we treated HSCs overexpressing ATG9b with inhibitors of the p38/JNK pathway or activators of the PI3K‐AKT pathway. The PI3K‐AKT pathway was mainly attributed to ACD rather than apoptosis in HSCs overexpressing ATG9b (Figure 6D,E). In contrast, the P38/JNK pathway was predominant in apoptosis rather than in the ACD of HSCs overexpressing ATG9b (Figure 6D,E). These results indicate that the ACD and apoptosis induced by ATG9b upregulation are independent of each other.

### 3.7 ATG9b attenuated liver fibrosis and promoted ACD of HSCs in vivo

**FIGURE 7:**
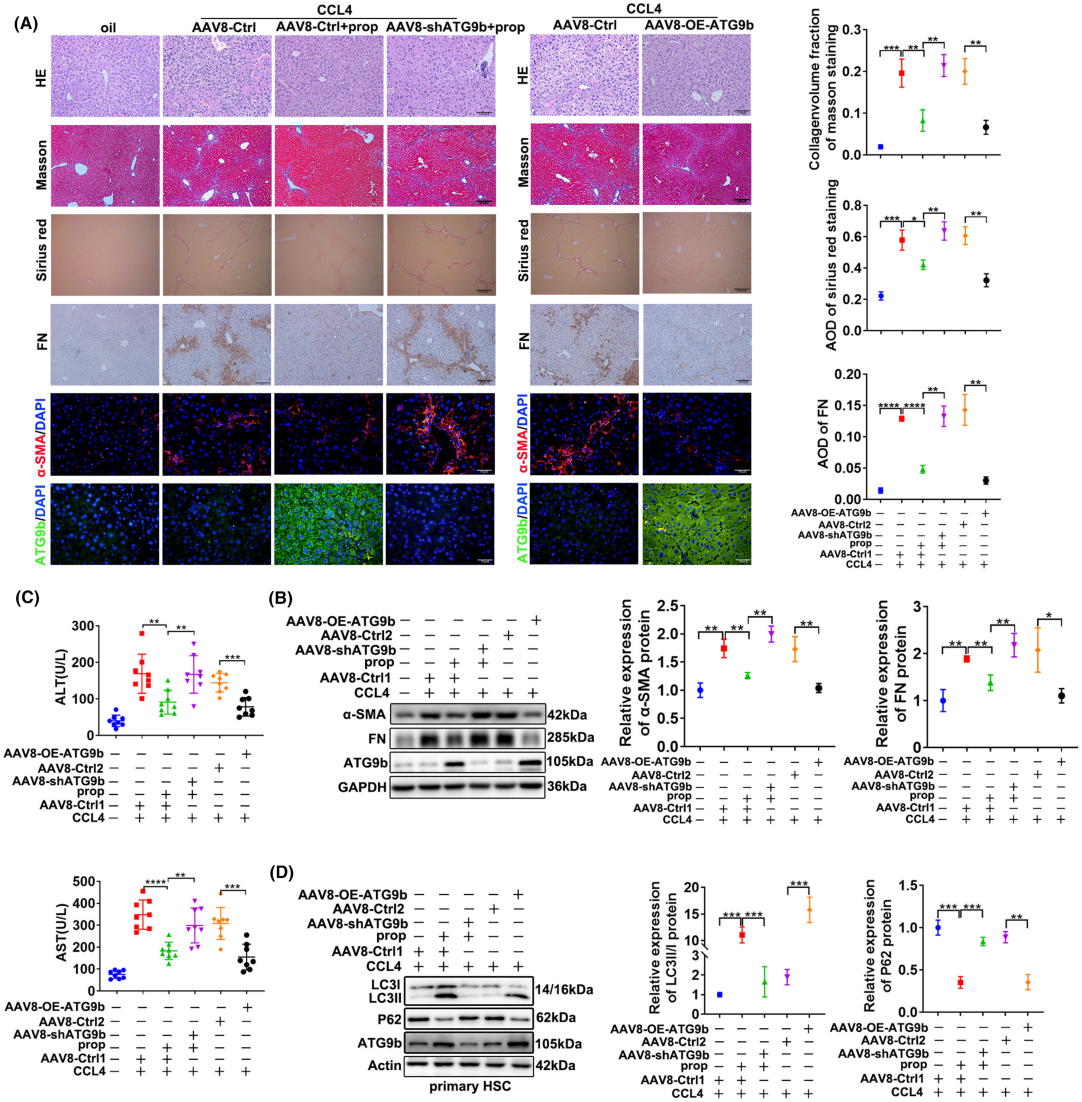
Upregulation of ATG9b attenuates liver fibrosis in vivo. (A) HE, Masson, Sirius red, immunohistochemical staining of FN and immunofluorescence of α‐SMA and ATG9b in liver tissues. (B) The α‐SMA and FN protein expression levels in liver tissues were determined using western blotting. (C) The levels of alanine aminotransferase (ALT) and aspartate aminotransferase (AST) in the different treatment groups. (D) Primary HSCs were extracted from different groups of mice. The protein levels of LC3B, P62 and ATG9b in primary HSCs were determined. Data are presented as mean ± standard error from three independent experiments (*p < 0.05, **p < 0.01, ***p < 0.001, ****p < 0.001).

We also explored the role of propranolol and ATG9b in liver fibrosis in vivo. The area of CCl_4_‐induced liver injury stained by HE, Masson and Sirius red was reduced following propranolol treatment, and the accumulation of FN and α‐SMA was reduced after propranolol treatment compared with CCl_4_ treatment alone. However, this protective effect was attenuated by ATG9b knockdown (Figure 7A). In addition, ATG9b overexpression protected mice from CCl_4_‐induced liver fibrosis (Figure 7A). The same outcome was confirmed by immunoblotting (Figure 7B) and serological analyses of ALT and AST levels (Figure 7C). Consistent with the in vitro experimental results, ATG9b knockdown reversed propranolol‐induced ACD of HSCs, and ATG9b upregulation resulted in the ACD of HSCs in cirrhotic mouse (Figure 7D). These results suggest that ATG9b may be a potential target candidate for inhibiting liver fibrosis.

## 4 DISCUSSION

Propranolol exhibits anti‐inflammatory, anti‐immune dysfunction and antiangiogenic effects, supporting its potentially beneficial role in liver fibrosis treatment.^24^, ^25^, ^26^ In this study, we confirmed that propranolol inhibits liver fibrosis in vivo and in vitro and first revealed that propranolol restrained the ability of activated HSCs by ACD. We found that preventing autophagy through CQ could reverse propranolol‐induced cell death, suggesting that the occurrence of ACD stimulated by propranolol causes HSC cell death. We then demonstrated that propranolol could increase autophagosome accumulation. The collection of autophagosomes may be the result of autophagy activation or failed clearance of autophagosomes. Therefore, the actual autophagic flux state remains unclear. Our study further provided strong evidence that propranolol promotes autophagic flux. However, the effect of autophagy on liver disease is controversial. On one hand, many previous studies showed that autophagy could promote HSC activation and aggravate liver fibrosis.^27^, ^28^, ^29^ On the other hand, some studies considered autophagy as a protective process for the liver.^30^, ^31^, ^32^ Consistent with our study, Zhang et al.^33^ found that β‐thujaplicin induces ACD through ROS‐mediated Akt signalling and alleviates human hepatocellular carcinoma. Thus, we speculate that autophagy in activated HSCs is a pathogenic mechanism of liver fibrosis and that ACD in activated HSCs acts as a protective mechanism of liver fibrosis.

This is the first study to reveal the role of ATG9b in liver fibrosis. Upregulation of ATG9b attenuated the activation of HSCs and improved CCl_4_‐induced liver fibrosis. We believe this alleviation may be attributed to the modulation of cell death in activated HSCs by ATG9b. ATG9 protein levels regulate the frequency of autophagosome formation, albeit with an unclear mechanism, and knockdown of ATG9 decreased the number of autophagic puncta, which reflects the influence of ATG9 proteins on autophagy.^34^, ^35^ A previous study demonstrated that ATG9b deficiency, but not that of its orthologue ATG9a, suppressed autophagy and potentiated hepatocyte apoptosis in hepatocarcinogenesis.^36^ It is the only essay explaining the different clinical values of ATG9 homologues in the liver. The study also indicates that the biological functions of ATG9a and ATG9b may be different from one another. Consistent with their results, we revealed that ATG9b overexpression in activated HSCs promoted the progression of autophagy. Another discrepant observation was that ATG9b overexpression potentiated apoptosis of activated HSCs, whereas ATG9b deficiency potentiated hepatocyte apoptosis in hepatocarcinogenesis. This difference may be because hepatocytes were more vulnerable to exacerbated ER stress and were susceptible to apoptosis when ATG9b deficiency caused damage in cytoprotective autophagy; nevertheless, ATG9b overexpression in activated HSCs led to ACD but not to cytoprotective autophagy. Thus, ATG9b may play various roles in different cells. Moreover, its protective function in hepatocytes and killing action in activated HSCs may act together to alleviate liver fibrosis. Whether ATG9b influences hepatocytes in liver fibrosis has yet to be studied. Further studies are required to explore these underlying mechanisms. Another limitation of this study is that ATG9b has not been studied in liver fibrosis in the clinics. Further studies are required to prove the role of ATG9b in fibrotic patients. Our findings also indicate that P62 deletion is likely due to increased protein aggregation in autophagic vacuoles in ATG9b‐overexpressing HSCs. Because there is no change in the interaction of the ATG5‐ATG12‐LC3 complex, increasing consumption of P62 may result from an increasing number of essential phagophores mediating the connection between P62 and autophagic membranes. Our study showed that ATG9b‐driven phagophores facilitate the association of LC3B to P62 and P62 to ubiquitinated substrates during autophagic protein degradation in activated HSCs because P62 has an LC3‐interaction region and a cargo‐binding element such as the Ub‐binding domains.^37^, ^38^

Furthermore, we explored the signalling pathways responsible for ACD and apoptosis in activated HSCs. The PI3K‐AKT‐mTOR and MAPK pathways play vital roles in cell motility.^39^, ^40^ Many studies showed that the PI3K‐AKT‐mTOR and MAPK pathways might crosstalk at various stages of signal transduction.^41^, ^42^, ^43^, ^44^ Surprisingly, our study showed that the PI3K‐AKT‐mTOR pathway regulated ACD without influencing apoptosis and that the MAPK pathway was involved in apoptosis without impacting ACD in ATG9b‐overexpressing HSCs. The results indicate that a change in the PI3K‐AKT‐mTOR pathway via activators affects apoptosis to a lesser extent than ATG9b overexpression and that the change in the MAPK pathway via inhibitors affects ACD to a lesser degree than ATG9b overexpression.

## 5 CONCLUSION

Propranolol induces ACD of activated HSCs to improve liver fibrosis in an ATG9b‐dependent manner. This study also elucidated the important role of ATG9b in liver fibrosis. ATG9b is upregulated in propranolol‐treated HSCs. Its downregulation abolished propranolol‐induced attenuation of liver fibrosis and HSC death, whereas its upregulation mediated the ACD of activated HSCs and alleviated liver fibrosis. Furthermore, the relationship between ATG9b and the death of activated HSCs is related to suppressing the PI3K‐Akt‐mTOR pathway and activating the p38/JNK MAPK pathway (Figure S5). Thus, this study expands our knowledge of regulating ACD in HSCs to improve liver fibrosis. Our findings also suggest the use of propranolol as a potential agent and *ATG9b* as a regulated gene for HSC ACD, which promotes the development of treatment against liver fibrosis.

## AUTHOR CONTRIBUTIONS

**Sining Wang:** Conceptualization (equal); data curation (lead); investigation (equal); methodology (equal); software (lead); writing – original draft (lead); writing – review and editing (equal). **Qian Ding:** Data curation (equal); funding acquisition (equal); investigation (equal); methodology (equal); software (equal); validation (equal). **Aiyuan Xiu:** Data curation (equal); formal analysis (equal); methodology (equal); software (equal); validation (equal). **Yifu Xia:** Data curation (equal); formal analysis (equal); investigation (equal); methodology (equal); software (equal). **Guangchuan Wang:** Conceptualization (equal); investigation (equal); methodology (equal); validation (equal). **Chunqing Zhang:** Conceptualization (lead); data curation (equal); funding acquisition (lead); investigation (equal); project administration (equal); writing – original draft (equal); writing – review and editing (lead).

## FUNDING INFORMATION

This study was supported by the National Natural Science Foundation of China (Grant No. 81970533 and 82000566).

## CONFLICT OF INTEREST STATEMENT

The authors confirm that there are no conflicts of interest.

## Supporting information


Figure S1

Figure S2

Figure S3

Figure S4

Figure S5

## Data Availability

The datasets used and/or analysed during the current study are available from the corresponding author on reasonable request.
