# Supplementary figures and images for: Upregulation of ATG9b by propranolol promotes autophagic cell death of hepatic stellate cells to improve liver fibrosis
Source: J Cell Mol Med. 2023 Nov 16;28(2):e18047. doi: 10.1111/jcmm.18047 (PMC10826435; doi:10.1111/jcmm.18047)

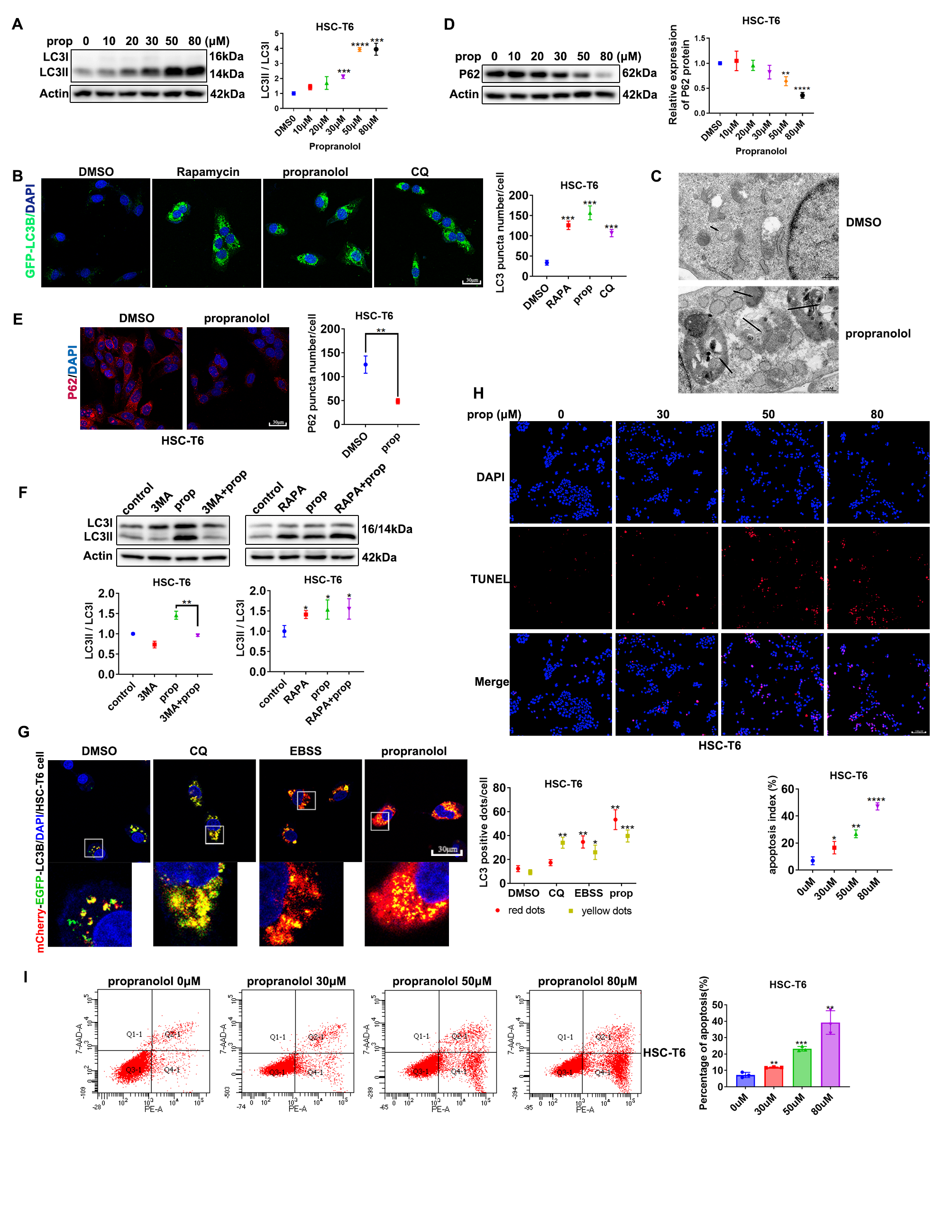

Supplement: Supplementary file 1 — Figure S1 [file JCMM-28-e18047-s002.tif]

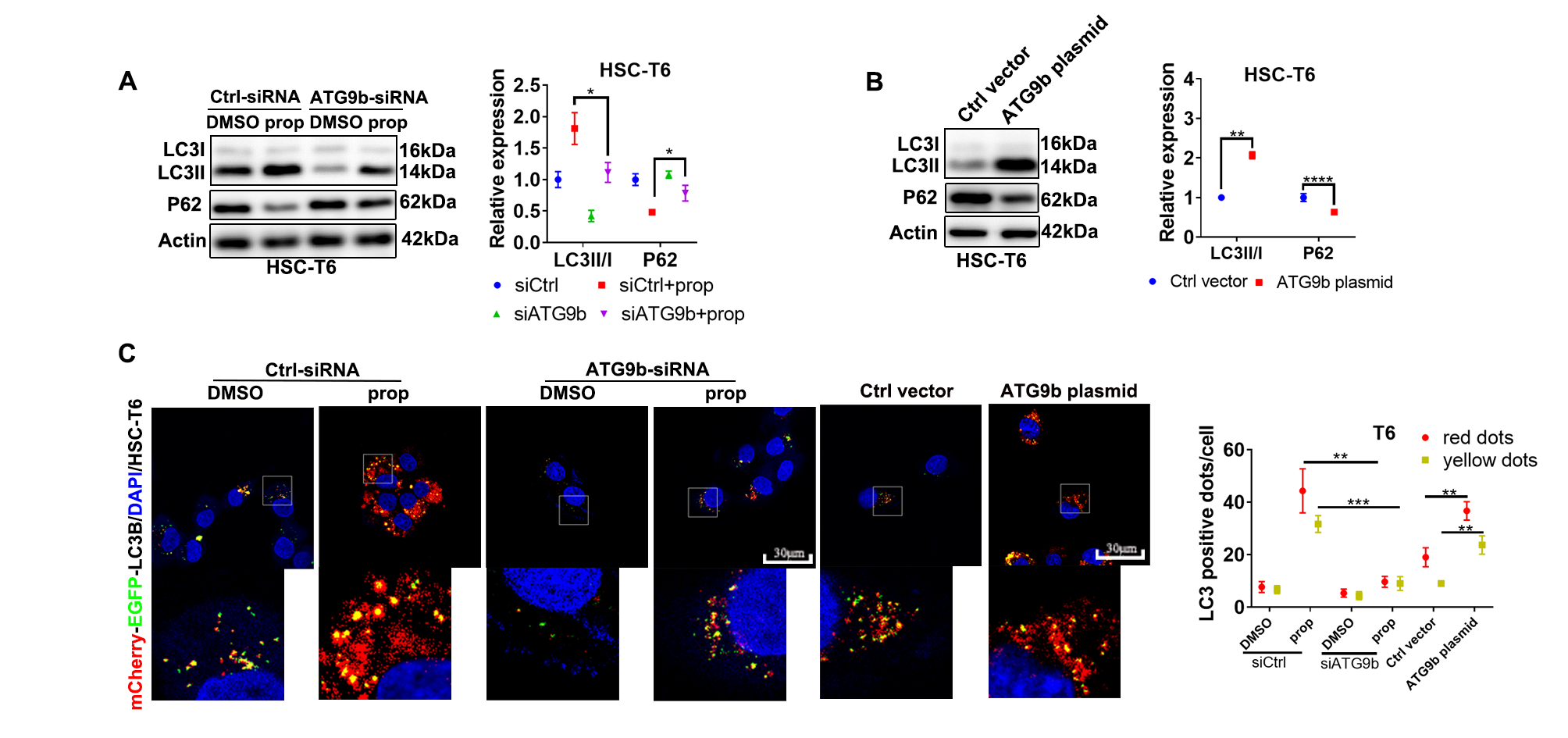

Supplement: Supplementary file 2 — Figure S2 [file JCMM-28-e18047-s001.tif]

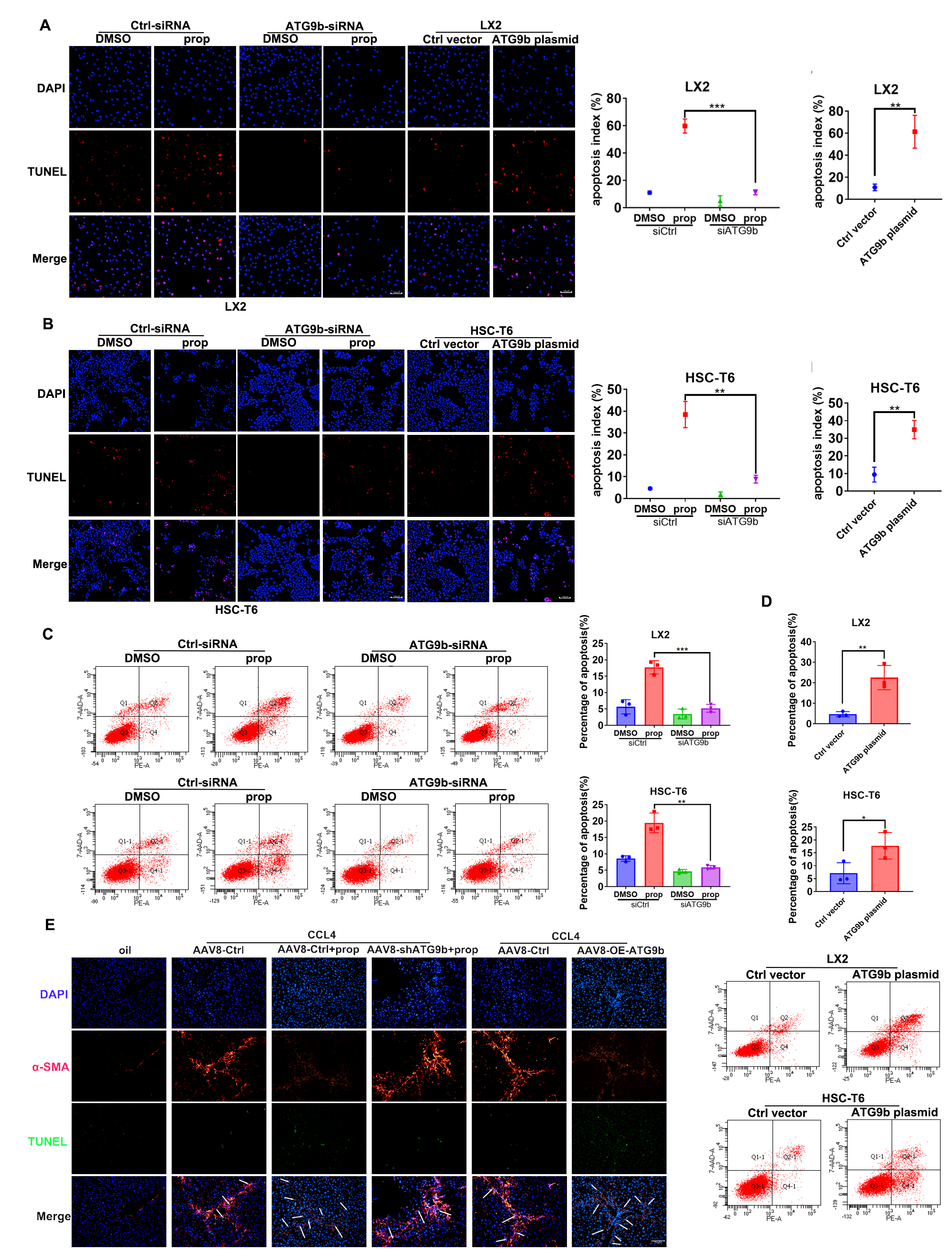

Supplement: Supplementary file 3 — Figure S3 [file JCMM-28-e18047-s005.tif]

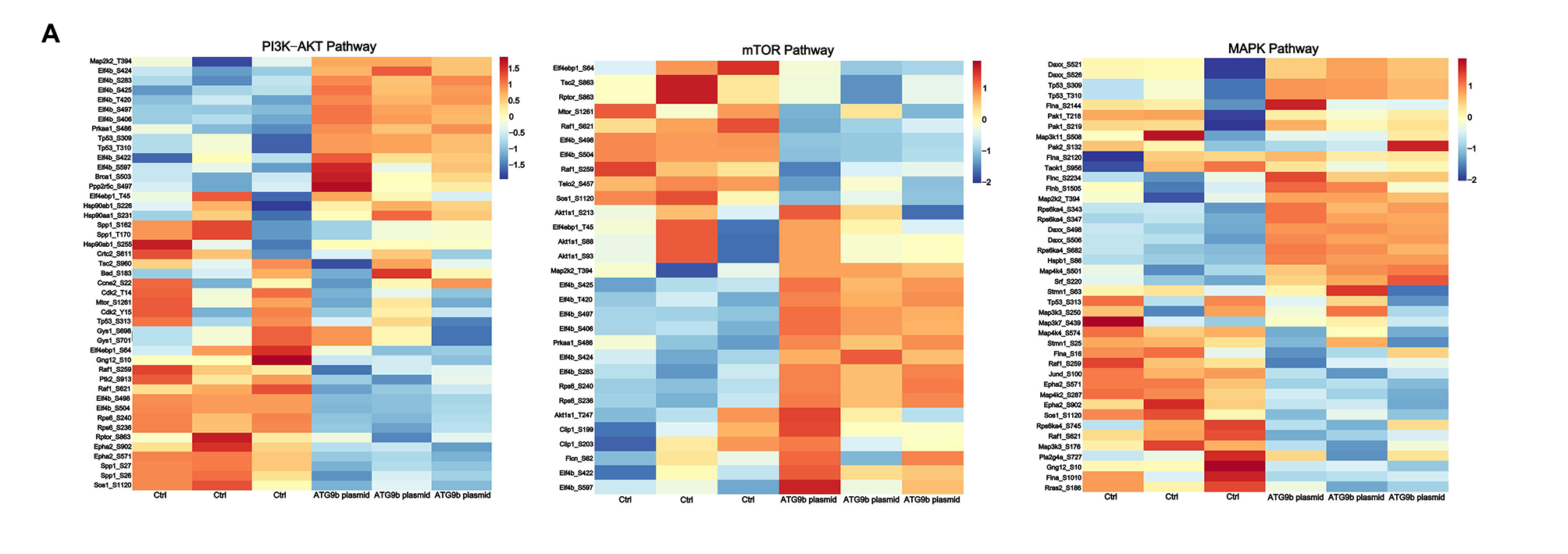

Supplement: Supplementary file 4 — Figure S4 [file JCMM-28-e18047-s003.tif]

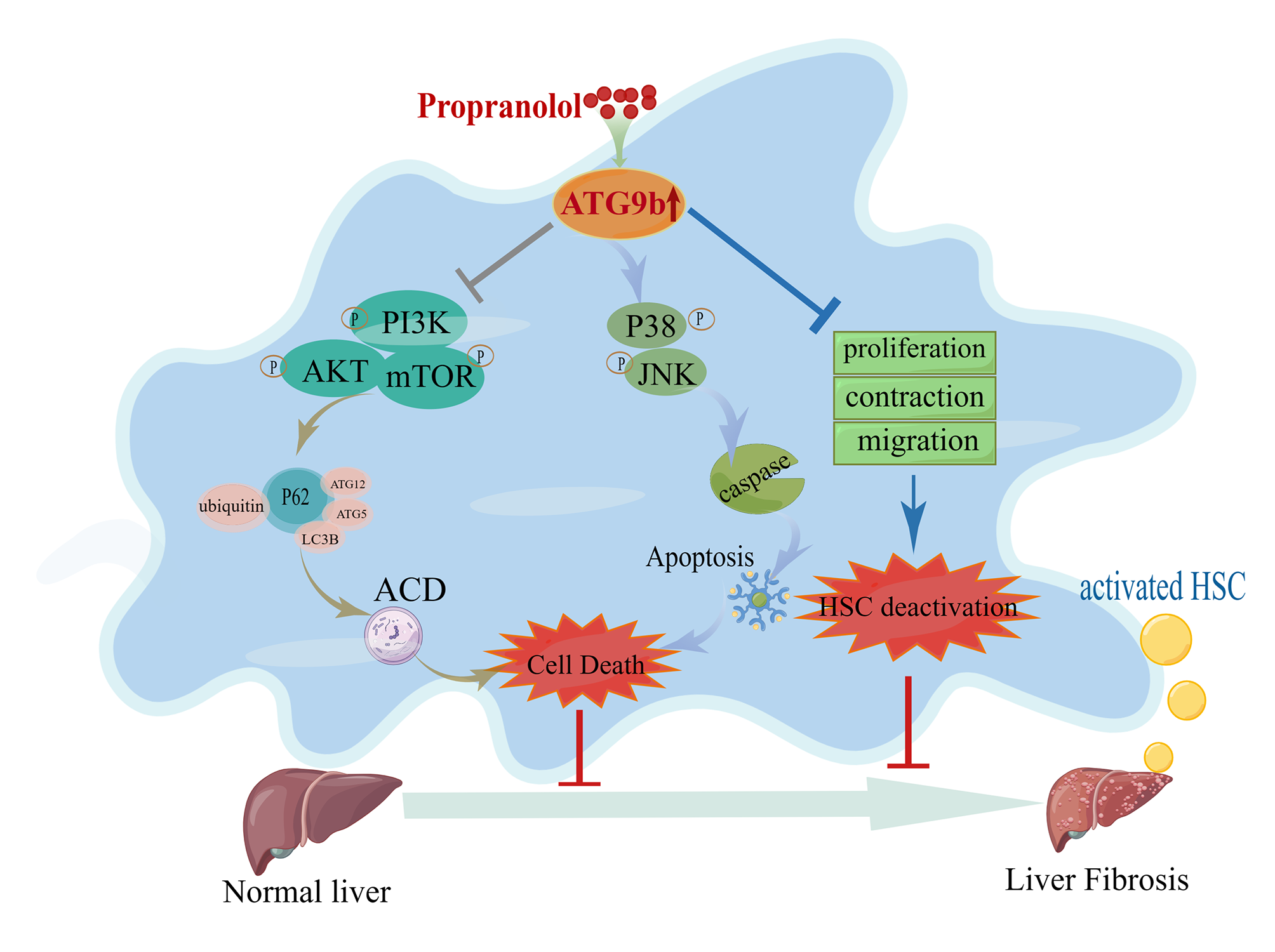

Supplement: Supplementary file 5 — Figure S5 [file JCMM-28-e18047-s004.tif]
